# Glycine and N‐acetylcysteine (GlyNAC) supplementation in older adults improves glutathione deficiency, oxidative stress, mitochondrial dysfunction, inflammation, insulin resistance, endothelial dysfunction, genotoxicity, muscle strength, and cognition: Results of a pilot clinical trial

**DOI:** 10.1002/ctm2.372

**Published:** 2021-03-27

**Authors:** Premranjan Kumar, Chun Liu, Jean W. Hsu, Shaji Chacko, Charles Minard, Farook Jahoor, Rajagopal V. Sekhar

**Affiliations:** ^1^ Translational Metabolism Unit, Division of Endocrinology, Diabetes and Metabolism Department of Medicine, Baylor College of Medicine Houston Texas 77030 USA; ^2^ USDA/ARS Children's Nutritional Research Center Houston Texas USA; ^3^ Institute of Clinical and Translational Research, Baylor College of Medicine Houston Texas

**Keywords:** aging, cognition, inflammation, insulin resistance, mitochondria, oxidative stress, strength

## Abstract

**Background:**

Oxidative stress (OxS) and mitochondrial dysfunction are implicated as causative factors for aging. Older adults (OAs) have an increased prevalence of elevated OxS, impaired mitochondrial fuel‐oxidation (MFO), elevated inflammation, endothelial dysfunction, insulin resistance, cognitive decline, muscle weakness, and sarcopenia, but contributing mechanisms are unknown, and interventions are limited/lacking. We previously reported that inducing deficiency of the antioxidant tripeptide glutathione (GSH) in young mice results in mitochondrial dysfunction, and that supplementing GlyNAC (combination of glycine and N‐acetylcysteine [NAC]) in aged mice improves naturally‐occurring GSH deficiency, mitochondrial impairment, OxS, and insulin resistance. This pilot trial in OA was conducted to test the effect of GlyNAC supplementation and withdrawal on intracellular GSH concentrations, OxS, MFO, inflammation, endothelial function, genotoxicity, muscle and glucose metabolism, body composition, strength, and cognition.

**Methods:**

A 36‐week open‐label clinical trial was conducted in eight OAs and eight young adults (YAs). After all the participants underwent an initial (pre‐supplementation) study, the YAs were released from the study. OAs were studied again after GlyNAC supplementation for 24 weeks, and GlyNAC withdrawal for 12 weeks. Measurements included red‐blood cell (RBC) GSH, MFO; plasma biomarkers of OxS, inflammation, endothelial function, glucose, and insulin; gait‐speed, grip‐strength, 6‐min walk test; cognitive tests; genomic‐damage; glucose‐production and muscle‐protein breakdown rates; and body‐composition.

**Results:**

GlyNAC supplementation for 24 weeks in OA corrected RBC‐GSH deficiency, OxS, and mitochondrial dysfunction; and improved inflammation, endothelial dysfunction, insulin‐resistance, genomic‐damage, cognition, strength, gait‐speed, and exercise capacity; and lowered body‐fat and waist‐circumference. However, benefits declined after stopping GlyNAC supplementation for 12 weeks.

**Conclusions:**

GlyNAC supplementation for 24‐weeks in OA was well tolerated and lowered OxS, corrected intracellular GSH deficiency and mitochondrial dysfunction, decreased inflammation, insulin‐resistance and endothelial dysfunction, and genomic‐damage, and improved strength, gait‐speed, cognition, and body composition. Supplementing GlyNAC in aging humans could be a simple and viable method to promote health and warrants additional investigation.

## Graphical abstract

This study found that compared to healthy young adults, older humans have severely elevated oxidative stress, glutathione deficiency, impaired mitochondrial function, increased inflammation, insulin resistance and endothelial dysfunction, and lower muscle strength and mental cognition. We tested and found that supplementing GlyNAC (combination of glycine and N‐acetylcysteine) improved all these defects, and that stopping GlyNAC resulted in a loss of benefits. The results of this trial suggests that GlyNAC supplementation could be a simple, safe and effective nutritional strategy to boost cellular defenses to protect against oxidative stress, correct mitochondrial defects to improve energy availability, increase muscle strength and cognition, and thereby promote healthy aging in humans.

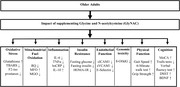

**Abbreviations**
8‐OHdG: 8‐hydroxy‐deoxyguanosine
AE: adverse effect
ALT: alanine transaminase
AST: aspartate transaminase
BDNF: brain‐derived neurotropic factor
BMI: body mass index
BUN: blood urea nitrogen
DEXA: dual‐energy x‐ray absorptiometry
DSST: digital symbol‐substitution test
GlyNAC: combination of glycine plus N‐acetylcysteine
GSH: glutathione
HbA1c: glycosylated hemoglobin
hsCRP: high‐sensitivity C‐reactive protein
IL‐10: interleukin‐10
IL‐6: interleukin‐6
IQR: interquartile range
MFO: mitochondrial fatty‐acid oxidation
MGO: mitochondrial glucose oxidation
MoCA: montreal cognitive assessment
MPBR: muscle protein breakdown rate
OA: older adults
OxS: oxidative stress
RBC: red‐blood cell
ROS: reactive‐oxygen species
sICAM1: soluble intracellular adhesion molecule 1
sVCAM1: soluble vascular‐cell adhesion molecule 1
TBARS: thiobarbituric acid reducing substances
YA: young adults

## 1 BACKGROUND

The free radical and the mitochondrial theories of aging suggest that elevated oxidative stress (OxS) and mitochondrial dysfunction^1^, ^2^ contribute to the aging process. Aging in older adults (OA) is associated with declining cognition,^3^, ^4^, ^5^ declining physical function,^6^, ^7^, ^8^ elevated inflammation,^9^, ^10^, ^11^, ^12^, ^13^ endothelial dysfunction,^14^ insulin resistance,^15^, ^16^ and central obesity.^17^, ^18^, ^19^ There is limited understanding as to why these defects occur in OA, and effective interventions for these defects are currently limited or lacking. Based on earlier published translational studies,^20^ we conducted an exploratory pilot open‐label clinical trial to test the effects of providing nutritional supplementation with GlyNAC (combination of glycine and N‐acetylcysteine) for a 24‐week duration on OxS, impaired mitochondrial fuel‐oxidation (MFO) in OA, inflammation, endothelial dysfunction, insulin‐resistance, muscle and glucose metabolism and assess the impact on cognition and physical function. The study also involved a 12‐week duration of GlyNAC withdrawal to study washout effects on several of these outcome measures.

Mitochondria are cellular engines which oxidize fatty‐acids and glucose to provide energy for cellular processes and thereby life. However, mitochondrial function is impaired in OA.^21^, ^22^, ^23^, ^24^, ^25^ Mitochondrial impairment has been linked to several age‐associated defects^26^ including elevated inflammation,^27^ insulin resistance,^28^, ^29^ endothelial dysfunction,^30^, ^31^, ^32^ physical decline,^33^, ^34^, ^36^ and cognitive impairment.^37^, ^38^, ^39^, ^40^, ^41^ During the process of fuel oxidation, mitochondria generate toxic reactive oxygen species (ROS) which results in harmful OxS, and therefore mitochondria depend on antioxidants for protection from the damaging effects of OxS. Glutathione (GSH) (γ‐glutamyl‐cysteinylglycine) is the most abundant endogenous, intracellular antioxidant composed of glycine, cysteine and glutamic acid. Aging is associated with GSH deficiency.^42^, ^43^, ^44^ We discovered and reported that the primary reason for GSH deficiency in OA is diminished synthesis caused by decreased availability of glycine and cysteine.^45^ Short‐term (2‐week) supplementation of GlyNAC corrected deficient GSH synthesis and concentrations and lowered ROS levels and damage caused by OxS to levels seen in younger humans.^45^ This study also answers the question of why GlyNAC is an improvement over supplementation with NAC alone. GSH synthesis requires two biochemical steps – in the first step cysteine is added to glutamic acid to form the intermediate glutamylcysteine, and in the second step glycine is added to glutamylcysteine to form GSH. Via translational studies in aged mice, we discovered and reported that GSH adequacy is critically important for optimal and efficient mitochondrial fatty‐acid oxidation (MFO) in rodents and OA.^20^ Because the impact of longer duration of supplementing GlyNAC in OA was unknown, we conducted a 36‐week open‐label trial to test whether supplementing GlyNAC for 24‐weeks in OA could improve or correct age‐associated GSH deficiency, OxS, impaired MFO, inflammation, endothelial dysfunction, insulin resistance, muscle protein loss, and body composition, and whether this could impact cognitive function, gait speed, muscle strength, and exercise capacity. The trial also assessed whether any accrued benefits would decline after stopping GlyNAC for 12 weeks.

## 2 METHODS

### 2.1 Study design

**FIGURE 1:**
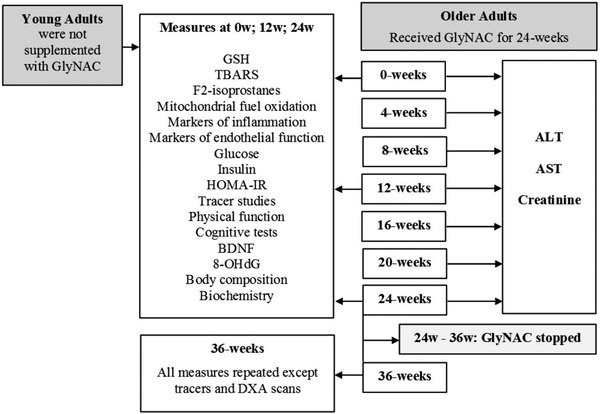
Study timeline. Abbreviations: ALT, alanine transaminase; AST, aspartate transaminase; BDNF, brain‐derived neurotropic factor; GlyNAC, combination of glycine and N‐acetylcysteine; GSH, glutathione; HOMA‐IR, homeostatic model assessment for insulin resistance; TBARS, thiobarbituric acid reducing substances; 8‐OHdG, 8‐hydroxy‐deoxyguanosine

An open‐label 36‐week clinical trial was approved by the Institutional Review Board at Baylor College of Medicine and registered with clinicaltrials.gov (NCT02348762) (Figure 1).

### 2.2 Participants

Eight OA (five women, three men; 71–80 years) and eight gender‐matched young adults (YAs) (21–30 years) who volunteered to participate in the study were recruited and enrolled after meeting inclusion/exclusion eligibility criteria. YAs were aged 24 ± 1.0 years, and OAs were aged 74 ± 1.0 years.

Inclusion criteria: Age range 70–80 years (OA), 20–30 years (YA). Exclusion criteria: Diabetes (fasting plasma glucose ≥ 126 mg/dl, HbA1c ≥ 6.5%), abnormal thyroid function tests, untreated thyroid or heart disease, active malignancy, liver or kidney impairment (alanine and aspartate transaminases [ALT and AST] > 2X upper limit of normal; serum creatinine > 1.5 mg/dl), anemia (hemoglobin < 11 g/dl), fasting triglycerides > 500 mg/dl, or inability to walk.

Non‐vitamin nutritional supplements or acetaminophen were stopped from 4 weeks prior to laboratory tests until the end of the 36‐week study. All participants were non‐smokers and asked to avoid consuming alcohol during the study. Participants were also asked to continue their usual habitual diets during the 36‐week period of the study. Because our exclusion criteria included individuals with cancer, diabetes, heart, liver, and renal diseases, medications were not a significant burden on these patients. None of the patients were taking any steroids or anti‐inflammatory agents.

### 2.3 Study protocol

Studies were conducted at the Metabolic Research Unit (MRU) at Baylor College of Medicine. There were two pre‐supplementation visits. Study participants first came to the MRU after an overnight fast for blood draws (plasma lipids, liver profile, blood urea nitrogen [BUN], Creatinine, thyroid stimulating hormone [TSH], free T4, cortisol, glucose, glycosylated hemoglobin [HbA1c], complete blood counts) and physical function assessment and were provided oral deuterated water (4 g/kg body weight) to be taken the night before the second visit. The participants then returned a few days later to undergo cognitive testing, dual‐energy x‐ray absorptiometry (DEXA) scan and metabolic studies. Intravenous catheters were placed in the dorsum of both hands. After basal blood sampling, tracers were infused (^2^H_2_‐glucose initiated at 21.6 μmol/kg, maintained at 21.6 μmol/kg/h for 5‐h; ^2^H_3_‐methylhistidine initiated at 0.06 μmol/kg, maintained at 0.03 μmol/kg/h for 3‐h). Blood was drawn during the final hour during steady‐state, centrifuged immediately and red‐blood cells (RBC) and plasma stored at −80°C for later analyses. Indirect calorimetry was performed for 45 min in the 5th h. Urine was collected for 6 h during the visit. Participants were discharged home after a meal. The YAs were not supplemented with GlyNAC and were released from the study. OAs were studied again at 12 weeks (midpoint of supplementation) to understand the impact of GlyNAC supplementation on GSH, OxS, MFO, physical and cognitive function, plasma biomarkers, and body‐composition, but tracer studies were not done at this time. At the end of the 24 weeks of supplementation, the full protocol (including tracer studies) was repeated. To determine the effects of stopping GlyNAC for 12 weeks on GSH, OxS, MFO, physical and cognitive function, and plasma biomarkers, OAs were studied again at 36‐weeks but participants did not have tracer infusions or DEXA scans at this time.

### 2.4 Supplement dosing and monitoring

OAs were provided capsules of glycine (1.33 mmol/kg/day) and cysteine (0.81 mmol/kg/day, provided as N‐acetylcysteine [NAC]) prepared by a licensed pharmacist, and replenished every 4‐weeks for 24‐weeks. Compliance with GlyNAC supplementation was assessed with phone calls and capsule‐counting every 4‐weeks when OA returned to collect GlyNAC capsules for the next 4‐weeks, and to have blood drawn for monitoring renal (creatinine) and liver function (as ALT and AST).

### 2.5 Outcome measures

#### 2.5.1 GSH concentrations and OxS

RBC total and reduced GSH concentrations were measured by liquid chromatography (Waters ACQUITY UPLC System), and concentrations of oxidized glutathione (GSSG) calculated as the difference between total and reduced GSH. TBARS (Cayman Chemical, Ann Arbor, MI, USA), and 8‐Iso‐Prostaglandin‐F2a (Cell Biolabs In., San Diego, CA) in plasma were measured as markers of OxS.

#### 2.5.2 Mitochondrial fuel oxidation

MFO was measured by calorimetry (Vmax Encore metabolic cart, CareFusion Inc., San Diego, CA) and reported as respiratory quotient, mitochondrial fatty‐acid, and glucose oxidation.^46^

#### 2.5.3 Insulin resistance

Fasting plasma glucose concentrations were measured with an automated glucose analyzer (YSI, Yellow Springs, OH) and insulin concentrations by ELISA kit (Mercodia, Uppsala, Sweden). Insulin resistance was calculated as HOMA‐IR using fasting plasma glucose and insulin concentrations, as reported by us previously.^20^

#### 2.5.4 Biomarkers of inflammation, endothelial dysfunction, and genomic damage

These biomarkers were measured using high‐sensitivity ELISA kits with measurement of IL6 human, TNFα, sICAM1, sVCAM1, and E‐selectin (Invitrogen, Thermo‐Scientific Inc., USA) and human C‐reactive protein (Quantikine ELISA kit, R&D Systems). The oxidative genomic (DNA/RNA) damage was measured using a high‐sensitivity ELISA Kit (Cayman Chemical Company, Ann Arbor, MI).

#### 2.5.5 Tracer studies (at 0 week and 24 weeks only)

(a) Muscle protein breakdown rate (MPBR): Stable isotope preparation: Sterile [^2^H_3_]‐3‐methylhistidine was purchased from Cambridge Isotopes Laboratories (Andover, MA, USA), dissolved in saline, and infused after sterility testing. Blood samples were centrifuged, and plasma was frozen until analysis.

Isotopic Analyses: serum isotope enrichment of 3‐methylhistidine was measured by liquid‐chromatography tandem mass‐spectrometry after conversion into its DANS (5‐[dimethylamino]‐1‐napthalene sulfonamide) derivative and analyzed using a Synergi Fusion‐RP 2.5μ 100 × 2.0 mm column (Phenomenex, Torrance, CA, USA) on a triple quadrupole mass‐spectrometer (TSQ Vantage; Thermo Scientific, San Jose, CA, USA). Ions m/z 403 and 406 to product ion m/z 170 for 3‐methylhistidine and [^2^H_3_]‐3‐methylhistidine were monitored. Instrumental control, data acquisition, and analysis were performed by the Xcalibur (version 2.1) software package (Thermo Scientific, San Jose, CA, USA). Serum 3‐methylhistidine concentration was measured in the basal sample by an isotope dilution method using [^2^H_3_]‐3‐methylhistidine as an internal standard. The samples were then analyzed as described above.

Calculations: 3‐methylhistidine (3MH) flux = F x [(IEi/IEp)‐1]; where F = isotope infusion rate; IEi = isotopic enrichment of the infusate in mol percent excess (MPE); IEp = isotopic enrichment at steady state in MPE.

MPBR: Because 3‐MH is released only from the breakdown of myofibrillar protein present in skeletal muscle, its flux is used to estimate MPBR based on a 3‐MH concentration of 3.64 μmol/g of muscle protein, that is, MPBR = 3MH flux/3.64.

(b) Rates of appearance of glucose, glucose production rate, gluconeogenesis, and glycogenolysis: Stable isotope preparation: Sterile and pyrogen free deuterium oxide (^2^H_2_O), ^2^H and [6,6‐^2^H2] glucose was purchased from Cambridge Isotopes Laboratories (Andover, MA). [6,6‐^2^H2] glucose was dissolved in isotonic water, filtered, and infused after sterility testing. Blood samples were centrifuged, and plasma was frozen.

Isotopic Analyses: The isotopic enrichment of [6,6‐^2^H_2_] glucose was measured by gas‐chromatography mass‐spectrometry (6890/5973 Agilent Technologies, Wilmington, DE, USA) using the penta‐acetate derivative. The incorporation of deuterium in glucose from ^2^H_2_O was determined using the average deuterium enrichment in glucose carbons 1, 3, 4, 5, and 6.^46^ Deuterium enrichment in plasma water was determined by Isotope Ratio Mass Spectrometry (Delta^+^XL IRMS Thermo Finnigan, Bremen, Germany).^47^, ^48^

Calculations: Rate of appearance of glucose (Ra glucose) = F x [(IEi/IEp)‐1], calculated during steady‐state from the M+2 enrichment of plasma [6,6‐^2^H_2_]‐glucose, where F = isotope infusion rate; IEi = isotopic enrichment of the infusate in mol percent excess (MPE); IEp = isotopic enrichment at steady state in MPE.

$$\begin{array}{ccc} & & \mathrm{Rate}\;\mathrm{of}\;\mathrm{glucose}\;\mathrm{production}\left(\mathrm{mg}/\mathrm{kg}/\min\right)\left(\mathrm{GPR}\right)=\\ & & \;\mathrm{Ra}\;\mathrm{glucose}-\mathrm{exogenous}\;\mathrm{glucose}\;\mathrm{tracer}\;\mathrm{infused} \end{array}$$

Fractional gluconeogenesis (GNG%) = ([M+1] [^2^H] [m/z 170/169] /6) / IE^2^H_2_O; where (M+1)(^2^H) (m/z 170/169) is the M+1 enrichment of deuterium in glucose measured using m/z 170/169, and "6" is the number of ^2^H labeling sites on the m/z 170/169 fragment of glucose (i.e., the average M+1 enrichment derived from deuterated water); IE^2^H_2_O is the deuterium enrichment in plasma water.

$$\begin{array}{ccc} & & \mathrm{Rate}\;\mathrm{of}\;\mathrm{gluconeogenesis}\left(\mathrm{mmol}/\mathrm{kg}/\min\right)=\\ & & \;\mathrm{Ra}\;\mathrm{glucose}\;x\;\mathrm{GNG}\%, \end{array}$$

$$\mathrm{Rate}\;\mathrm{of}\;\mathrm{glycogenolysis}\left(\mathrm{mmol}/\mathrm{kg}/\min\right)=\mathrm{GPR}-\mathrm{GNG}\%$$

#### 2.5.6 Physical function

We used standard tests of physical function which have been used and validated in the clinical out‐patient setting in OA. These include gait speed (10 m walk, with subject walking comfortably), grip strength (best of 3 readings using a Jamar dynamometer), and exercise capacity (rapid 6‐min walk test).

#### 2.5.7 Cognitive function

Participants in this study did not have a known diagnosis of abnormal cognition. The cognitive status of participants was assessed using tests which are commonly used in the clinical outpatient setting to test cognition in OA and include the Montreal cognitive assessment (MoCA), trail‐making tests A and B, verbal fluency test, and the digital‐symbol substitution test (DSST).

#### 2.5.8 Body composition and anthropometry

Total body fat and truncal fat were measured by DEXA scans. Height and weight were measured using a stadiometer and calibrated weighing scale, respectively and used to calculate body mass index. Waist circumference was measured using a tape measure, and the same sites were used for repeat measurements.

#### 2.5.9 Plasma biochemistry

Fasting plasma concentrations of liver profile, lipid profile, Creatinine, BUN, HbA1c, cortisol, Free T4, and TSH were measured at the Clinical Pathology Labs.

### 2.6 Statistics

Baseline characteristics are summarized by means with standard deviations. An unpaired *t*‐test was used to compare OA and YAs in the pre‐supplemented state. A general linear mixed model was used to test for changes in outcomes across time points for OA. The model included fixed effects for study time (discrete) and the matrix of correlated residuals assumed an unstructured format. The mixed model is used to estimate means with 95% confidence intervals, and test the null hypothesis that there is no difference in means between time points. All hypothesis tests were assessed at the 0.05 level (two‐sided). *p*‐values were not adjusted for multiple hypothesis testing due to the small sample size of this pilot study.

## 3 RESULTS

### 3.1 Adverse effects and subject withdrawals

All participants completed the study, and there were no withdrawals from the study. An adverse event (AE) was defined as any side effect of GlyNAC. Any event that was reported to either the study team or research nurse by the subject was documented as such. The following grading scale for AEs was used:

0 = No AE; 1 = Mild AE requiring no intervention; 2 = Moderate AE requiring treatment; 3 = Severe AE requiring admission to a hospital; 4 = Life‐threatening AE; 5 = Death. The AE score was 0 as there were no AEs reported. Monitoring plasma creatinine, alanine transaminase, and aspartate transaminase every 4‐weeks did not show any elevations or abnormalities.

### 3.2 Plasma biochemistry and monitoring of liver transaminases and creatinine

Participants had normal pre‐supplementation laboratory results as shown in Table 1.

**TABLE 1:** Fasting plasma biochemistry. Values are means ± SD, and medians (interquartile range [IQR]). Means are significantly different at *p* < 0.05

Parameters	Young adults: 0‐week	Older adults: 0‐week	Older adults: after 24‐weeks on GlyNAC
		*YA v OA 0‐week*	*OA‐0‐week v OA‐24‐weeks*
Hemoglobin (g/L)	14.1 ± 0.8	13.5 ± 0.9	13.3 ± 0.5
	13.9 (1.0)	13.9 (1.1)	13.2 (0.7)
		*p = 0.66*	*p = 0.47*
Total protein (g/dl)	7.5 ± 0.4	7.0 ± 0.4	6.5 ± 0.3
	7.4 (0.8)	7.0 (0.5)	6.4 (0.5)
		*p = 0.018*	*p = 0.0006*
Total bilirubin (mg/dl)	0.6 ± 0.2	0.7 ± 0.3	0.6 ± 0.2
	0.6 (0.3)	0.8 (0.5)	0.6 (0.2)
		*p = 0.29*	*p = 0.12*
Alanine transaminase (U/L)	17.8 ± 4.8	17.6 ± 2.9	16.9 ± 4.7
	16.0 (6.0)	17.5 (3.5)	18.0 (4.0)
		*p = 0.96*	*p = 0.41*
Aspartate transaminase (U/L)	21.3 ± 5.3	21.0 ± 4.1	21.3 ± 4.9
	20.5 (6.0)	19.5 (5.0)	21.0 (5.5)
		*p = 0.88*	*p = 0.84*
Alkaline phosphatase (U/L)	60.8 ± 14.7	79.1 ± 30.7	87.4 ± 35.3
	62.5 (24.5)	65.0 (44.0)	79.0 (48.0)
		*p = 0.11*	*p = 0.051*
BUN (mmol/L)	12.1 ± 3.6	15.3 ± 3.9	11.6 ± 4.2
	11.0 (5.5)	14.5 (3.5)	10.5 (3.5)
		*p = 0.13*	*p = 0.017*
Creatinine (mg/dl)	0.8 ± 0.2	0.9 ± 0.3	0.8 ± 0.3
	0.8 (0.2)	0.9 (0.3)	0.7 (0.4)
		*p = 0.065*	*p = 0.022*
Estimated GFR	105.0 ± 21.3	73.1 ± 13.0	84.5 ± 16.1
	107 (37)	72.0 (20.0)	87.5 (23.5)
		*p = 0.005*	*p = 0.024*
HbA1c (%)	5.3 ± 0.3	5.7 ± 0.3	5.6 ± 0.3
	5.3 (0.4)	5.8 (0.5)	5.6 (0.5)
		*p = 0.059*	*p = 0.41*
Total cholesterol (mg/dL)	155.4 ± 10.3	225.4 ± 43.7	215.1 ± 54.9
	152.5 (14.0)	225.0 (31.0)	215.5 (45.5)
		*p = 0.003*	*p = 0.13*
Triglycerides (mg/dL)	63.5 ± 19.6	153.3 ± 91.7	105.3 ± 52.1
	60.5 (17.5)	131.0 (133.5)	93.5 (32.0)
		*p = 0.042*	*p = 0.048*
HDL‐cholesterol (mg/dL)	63.5 ± 18.7	62.4 ± 24.0	60.4 ± 24.9
	62.0 (23.5)	53.5 (31.5)	56.0 (36.0)
		*p = 0.92*	*p = 0.35*
LDL‐cholesterol (mg/dL)	79.0 ± 16.5	134.9 ± 22.3	133.8 ± 39.1
	81.5 (23.0)	139.0 (28.0)	131.0 (48.5)
		*p = 0.0009*	*p = 0.89*
Non‐HDL cholesterol (mg/dL)	90.6 ± 20.9	163.0 ± 37.0	154.8 ± 43.4
	93.0 (35.0)	166.5 (50.0)	148.5 (62.0)
		*p = 0.0095*	*p = 0.12*
TSH (mIU/L)	2.2 ± 1.0	2.3 ± 1.2	1.9 ± 1.3
	1.9 (1.5)	2.3 (1.9)	1.7 (0.9)
		*p = 0.90*	*p = 0.35*
Free T4 (ng/L)	1.2 ± 0.1	1.2 ± 0.1	1.2 ± 0.1
	1.2 (0.2)	1.1 (0.2)	1.2 (0.1)
		*p = 0.34*	*p = 1.0*

### 3.3 GSH and OxS

Compared to YA controls, RBC concentrations of reduced‐GSH in OA were 76% lower, and plasma TBARS and F2‐isoprostane concentrations were 845% and 318% higher, respectively (Table 2) (Figure 2). GlyNAC supplementation was associated with a 200% increase in RBC concentrations of reduced‐GSH, and 75% and 74% decline in TBARS and F2‐isoprostance concentrations respectively. Benefits receded on stopping GlyNAC with a 67% decline in RBC reduced‐GSH, 219% increase in TBARS, and 26% increase in F2‐isoprostane concentrations.

**TABLE 2:** Effect of GlyNAC supplementation and withdrawal on GSH and oxidative stress. OA received GlyNAC supplementation for 24‐weeks, and GlyNAC was stopped for 12 weeks from week 24 to week 36. Data are reported as means ± SD and median (IQR). Means are significantly different at *p* < 0.05

Outcome measure	Young adults: 0‐week	Older adults: 0‐week	Older adults: after 12‐weeks on GlyNAC	Older adults: after 24‐weeks on GlyNAC	Older adults: 36‐weeks (12‐weeks after stopping GlyNAC)
		*YA v OA 0‐week*	*OA‐0‐week v OA‐12‐weeks*	*OA‐0‐week v OA‐24‐weeks*	*OA‐24‐weeks v OA‐36‐weeks*
RBC‐total GSH (mmol/L.RBC)	1.8 ± 0.3	0.8 ± 0.4	1.0 ± 0.3	1.6 ± 0.2	0.9 ± 0.3
	1.8 (0.4)	0.7 (0.7)	1.0 (0.4)	1.6 (0.3)	0.9 (0.4)
		*p = 0.0005*	*p = 0.0045*	*p = 0.0004*	*p = 0.0029*
RBC‐reduced GSH (mmol/L.RBC)	1.7 ± 0.2	0.4 ± 0.2	0.7 ± 0.4	1.2 ± 0.1	0.4 ± 0.2
	1.7 (0.3)	0.4 (0.3)	0.7 (0.5)	1.1 (0.2)	0.4 (0.3)
		*p = 0.0000*	*p = 0.043*	*p = 0.0000*	*p = 0.0001*
RBC‐GSSG (mmol/L.RBC)	0.2 ± 0.1	0.4 ± 0.3	0.4 ± 0.4	0.4 ± 0.2	0.4 ± 0.2
	0.1 (0.1)	0.3 (0.5)	0.2 (0.3)	0.4 (0.3)	0.4 (0.2)
		*p = 0.026*	*p = 0.91*	*p = 0.96*	*p = 0.73*
RBC GSH/GSSG	10.1 ± 2.9	1.1 ± 0.8	3.4 ± 2.7	4.7 ± 3.0	1.1 ± 0.4
	9.7 (1.6)	0.7 (0.6)	3.4 (5.1)	3.7 (4.3)	1.0 (0.4)
		*p = 0.0003*	*p = 0.095*	*p = 0.022*	*p = 0.011*
Plasma TBARS (μM/L)	2.4 ± 0.4	22.7 ± 3.5	14.1 ± 6.0	5.7 ± 1.8	18.2 ± 2.2
	2.5 (0.5)	23.8 (5.1)	13.8 (5.8)	4.9 (1.8)	17.3 (1.9)
		*p = 0.0000*	*p = 0.0008*	*p = 0.0000*	*p = 0.0000*
Plasma F2‐isoprostane (pg/ml)	44.5 ± 7.3	185.9 ± 43.5	64.4 ± 8.3	47.8 ± 7.4	60.2 ± 9.1
	43.8 (6.9)	190.1 (45.5)	65.3 (12.2)	45.4 (9.7)	58.8 (12.1)
		*p = 0.0001*	*p = 0.0001*	*p = 0.0000*	*p = 0.017*

FIGURE 2Box plots showing the effects of GlyNAC supplementation and withdrawal on outcomes measures in young (Y) versus older adults (OA) at baseline (0 week), midpoint (12 weeks), completion of supplementation (24 weeks), and after 12 weeks of stoppage (36 weeks). (A) RBC‐total glutathione concentrations; (B) plasma TBARS concentrations; (C) mitochondrial fatty‐acid oxidation (whole‐body); (D) mitochondrial glucose oxidation (whole‐body); (E) plasma high‐sensitivity Interleukein‐6 (hsIL6) concentrations; (F) plasma soluble vascular cell adhesion molecule 1 (sVCAM1) concentrations; (G) insulin resistance (as HOMA‐IR); (H) gait speed; (I) trail‐making test B (TMT‐B); (J) plasma BDNF concentrations

**Figure:**
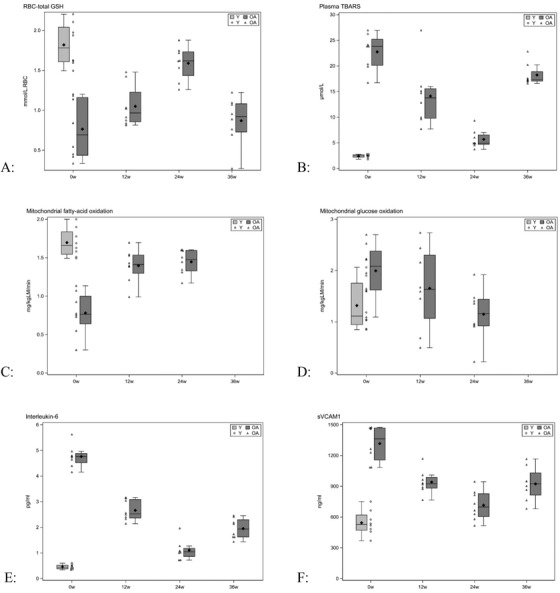

**Figure:**
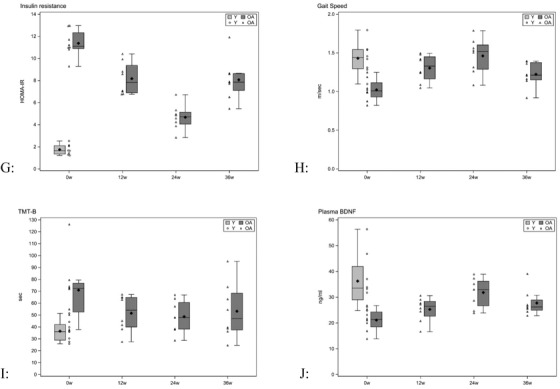

### 3.4 Mitochondrial fuel oxidation

Compared to fasting YAs, fasting OA had a significantly higher respiratory quotient and abnormal mitochondrial fuel oxidation, with a significant 54% lower mitochondrial fatty‐acid oxidation and 51% higher MGO (Table 3) (Figure 2). GlyNAC supplementation corrected defective MFO (in mitochondrial fatty‐acid and glucose oxidation) but did not affect energy expenditure. We did not measure body composition after stopping GlyNAC, but the respiratory quotient showed an increase suggesting recurrence of mitochondrial fuel impairment after discontinuing GlyNAC.

**TABLE 3:** Effect of GlyNAC supplementation and withdrawal on mitochondrial fuel oxidation, respiratory quotient, and energy expenditure. OA received GlyNAC supplementation for 24 weeks, and GlyNAC was stopped for 12 weeks from week 24 to Week 36. Data are reported as means ± SD and median (IQR). Means are significantly different at *p* < 0.05

Outcome measure	Young adults: 0‐week	Older adults: 0‐week	Older adults: after 12‐weeks on GlyNAC	Older adults: after 24‐weeks on GlyNAC	Older adults: 36‐weeks (12‐weeks after stopping GlyNAC)
		*YA v OA 0‐week*	*OA‐0‐week v OA‐12‐weeks*	*OA‐0‐weeks v OA‐24‐weeks*	*OA‐24‐weeks v OA‐36‐weeks*
Fasting respiratory quotient (RQ)	0.77 ± 0.01	0.84 ± 0.03	0.79 ± 0.04	0.77 ± 0.03	0.83 ± 0.03
	0.76 (0.0)	0.86 (0.0)	0.80 (0.1)	0.78 (0.0)	0.84 (0.0)
		*p = 0.0008*	*p = 0.02*	*p = 0.0000*	*p = 0.0003*
Fasting mitochondrial fatty‐acid oxidation (mg/kgLM/min)	1.7 ± 0.2	0.8 ± 0.3	1.4 ± 0.2	1.5 ± 0.2	
	1.7 (0.3)	0.8 (0.4)	1.4 (0.2)	1.5 (0.3)	
		*p = 0.0004*	*p = 0.0018*	*p = 0.0002*	
Fasting mitochondrial carbohydrate oxidation (mg/kgLM/min)	1.3 ± 0.5	2.0 ± 0.5	1.7 ± 0.8	1.2 ± 0.5	
	1.1 (0.8)	2.1 (0.8)	1.6 (1.2)	1.2 (0.5)	
		*p = 0.034*	*p = 0.26*	*p = 0.0026*	
Energy expenditure (kcal/d)	1530 ± 284	1319 ± 198	1280 ± 212	1291 ± 205	1252 ± 249
	1535 (355)	1294 (367)	1202 (247)	1269 (262)	1242 (229)
		*p = 0.041*	*p = 0.51*	*p = 0.63*	*p = 0.65*

### 3.5 Inflammation, endothelial function, glycemic and genotoxicity measures

Compared to YAs, OA had significantly elevated plasma levels of pro‐inflammatory cytokines (interleukin‐6, IL‐6; tumor necrosis factor alpha, TNFα; high‐sensitivity C‐reactive protein, hsCRP) and decreased levels of an anti‐inflammatory cytokine interleukin‐10 (IL‐10) (Table 4) (Figure 2). Fasting plasma concentrations of IL‐6 were 934% higher, TNFα 116% higher, hsCRP 88% higher, and IL‐10 29% lower. Endothelial function was measured as plasma biomarkers sVCAM1 (soluble vascular cell adhesion molecule‐1), sICAM1 (soluble intercellular adhesion molecule‐1), and E‐selectin. Fasting plasma sVCAM1 levels were 175% higher, sICAM1 142% higher, and E‐selectin 63% higher. Fasting plasma concentrations of glucose and insulin were 15% and 469% higher, respectively, and insulin resistance was 571% higher. Plasma concentrations of 8‐hydroxy‐deoxyguanosine (8‐OHdG), a marker of DNA damage, were significantly higher by 348%. GlyNAC supplementation for 24 weeks lowered IL‐6, TNFα, hsCRP by 77, 57% and 49%; increased IL‐10 by 38%; decreased sICAM1, sVCAM1 and E‐selectin by 60%, 46% and 35%; significantly lowered fasting plasma glucose (9% lower), insulin (55% lower) and insulin resistance (59% lower); and lowered 8OHdG by 66%. Stopping GlyNAC led to a reversal of accrued benefits in all parameters as shown in Table 4, suggesting that continued GlyNAC supplementation may be required to maintain benefits.

**TABLE 4:** Effect of GlyNAC supplementation and withdrawal on inflammation, endothelial function, glycemic indices, and tracer data. OA received GlyNAC supplementation for 24 weeks, and GlyNAC was stopped for 12 weeks from week 24 to week 36. Data are reported as means ± SD and median (IQR). Means are significantly different at *p* < 0.05

Outcome measures	Young adults: 0‐week	Older adults: 0‐week	Older adults: after 12‐weeks on GlyNAC	Older adults: after 24‐weeks on GlyNAC	Older adults: 36‐weeks (12‐weeks after stopping GlyNAC)
		*YA v OA 0‐week*	*OA‐0‐week v OA‐12‐weeks*	*OA‐0‐week v OA‐24‐weeks*	*OA‐24‐weeks v OA‐36‐weeks*
**Plasma markers of inflammation**					
IL‐6 (pg/ml)	0.5 ± 0.1	4.8 ± 0.4	2.7 ± 0.4	1.1 ± 0.4	2.0 ± 0.4
	0.4 (0.1)	4.8 (0.4)	2.5 (0.7)	1.0 (0.3)	1.9 (0.7)
		*p = 0.0000*	*p = 0.0000*	*p = 0.0000*	*p = 0.0001*
TNFα (pg/ml)	45.3 ± 9.4	97.9 ± 13.9	80.4 ± 10.6	58.7 ± 8.3	72.6 ± 11.1
	45.0 (15.1)	97.9 (23.7)	80.5 (12.7)	57.2 (9.0)	71.4 (16.1)
		*p = 0.0000*	*p = 0.0000*	*p = 0.0000*	*p = 0.0003*
High sensitivity C‐reactive protein (hsCRP, ng/ml)	2.4 ± 0.4	4.9 ± 0.6	3.5 ± 0.5	3.2 ± 0.5	3.9 ± 0.5
	2.5 (0.3)	4.8 (0.6)	3.5 (0.9)	3.0 (0.8)	3.8 (0.6)
		*p = 0.0000*	*p = 0.0002*	*p = 0.0000*	*p = 0.0004*
IL‐10 (pg/ml)	3.5 ± 1.1	2.5 ± 0.6	2.8 ± 0.5	3.4 ± 0.4	2.6 ± 0.5
	3.4 (2.0)	2.3 (1.0)	2.6 (0.8)	3.5 (0.6)	2.6 (0.7)
		*p = 0.029*	*p = 0.0005*	*p = 0.0000*	*p = 0.0002*
**Plasma markers of endothelial function**					
Plasma sICAM1 (ng/ml)	382 ± 64	1054 ± 173	507 ± 125	421 ± 75	510 ± 56
	399 (91)	1099 (340)	513 (177)	427 (81)	499 (69)
		*p = 0.0000*	*p = 0.0002*	*p = 0.0000*	*p = 0.0007*
Plasma sVCAM1 (ng/ml)	545 ± 120	1317 ± 173	941 ± 117	716 ± 145	924 ± 162
	528 (148)	1363 (312)	926 (104)	698 (223)	926 (216)
		*p = 0.0000*	*p = 0.0003*	*p = 0.0002*	*p = 0.0001*
Plasma E‐selectin (ng/ml)	7.5 ± 1.7	12.2 ± 1.6	10.5 ± 1.6	8.0 ± 1.4	9.7 ± 1.2
	7.7 (2.5)	12.4 (2.1)	10.6 (2.2)	7.5 (2.0)	9.8 (1.5)
		*p = 0.0002*	*p = 0.0002*	*p = 0.0000*	*p = 0.0009*
**Fasting glycemic indices**					
Plasma glucose (mmol/L)	4.7 ± 0.1	5.4 ± 0.9	5.1 ± 0.8	4.9 ± 0.6	5.4 ± 0.6
	4.7 (0.2)	5.3 (1.1)	5.1 (0.9)	4.8 (0.7)	5.3 (0.8)
		*p = 0.034*	*p = 0.18*	*p = 0.04*	*p = 0.047*
Plasma insulin (mU/L)	8.4 ± 2.4	47.8 ± 6.3	36.1 ± 4.1	21.4 ± 3.8	33.5 ± 6.5
	7.8 (3.8)	47.5 (6.4)	35.4 (3.2)	22.7 (6.6)	32.2 (9.7)
		*p = 0.0000*	*p = 0.0000*	*p = 0.0000*	*p = 0.0004*
Insulin resistance (HOMA‐IR)	1.7 ± 0.5	11.4 ± 1.2	8.2 ± 1.5	4.7 ± 1.1	8.1 ± 1.9
	1.7 (0.8)	11.1 (1.4)	7.8 (2.5)	4.7 (1.1)	7.9 (1.5)
		*p = 0.0000*	*p = 0.0000*	*p = 0.0000*	*p = 0.0008*
**Fasting tracer data**					
Rate of glucose production (μmol/kg/min)	9.3 ± 1.1	8.8 ± 0.8		8.8 ± 1.1	
	9.6 (1.8)	8.8 (1.3)		9.3 (1.6)	
		*p = 0.18*		*p = 0.66*	
Rate of glycogenolysis (μmol/kg/min)	3.0 ± 0.9	3.0 ± 0.7		2.9 ± 0.7	
	2.6 (1.7)	3.2 (1.2)		2.8 (1.1)	
		*p = 0.91*		*p = 0.48*	
Rate of gluconeogenesis (μmol/kg/min)	6.1 ± 0.9	5.4 ± 0.5		5.6 ± 0.8	
	6.2 (1.4)	5.3 (0.6)		5.4 (1.4)	
		*p = 0.13*		*p = 0.16*	
Muscle breakdown rate (μmol/kg/min)	120.3 ± 32.5	162.5 ± 52.6		128.9 ± 29.4	
	129.3 (54.2)	145.7 (58.8)		131.7 (34.9)	
		*p = 0.11*		*p = 0.0079*	
**Genomic damage**					
8‐hydroxy deoxyguanosine (pg/ml)	23.9 ± 3.8	106.9 ± 30.1	59.7 ± 16.2	36.9 ± 11.6	50.1 ± 17.6
	23.3 (6.0)	120.4 (37.3)	60.2 (13.5)	37.5 (14.6)	50.3 (24.4)
		*p = 0.000095*	*p = 0.0002*	*p = 0.0002*	*p = 0.026*

### 3.6 Tracer studies

Compared to YAs, the rate of muscle protein breakdown in OA was 35% higher, and this declined by 21% with GlyNAC supplementation (Table 4). Rates of glucose production, gluconeogenesis, and glycogenolysis did not show any differences between YAs and OA in the pre‐supplemented state and did not change with GlyNAC supplementation.

### 3.7 Body weight, BMI, total body fat, truncal fat, and waist circumference

OA had significantly higher total body weight (8.7% higher), BMI (10.6% higher), total body fat (47.7% higher), and waist circumference (12 cm higher) (Table 5). GlyNAC supplementation for 24 weeks was associated with significant decreases in fat mass (4% lower) and waist circumference (4 cm lower). These benefits began to reverse on stopping GlyNAC.

**TABLE 5:** Effect of GlyNAC supplementation and withdrawal on body composition. OA received GlyNAC supplementation for 24‐weeks, and GlyNAC was stopped for 12 weeks from week 24 to week 36. Data are reported as means ± SD and median (IQR). Means are significantly different at *p* < 0.05

Body composition	Young adults: 0‐week	Older adults: 0‐week	Older adults: after 12‐weeks on GlyNAC	Older adults: after 24‐weeks on GlyNAC	Older adults: 36‐weeks (12‐weeks after stopping GlyNAC)
		*YA v OA 0‐week*	*OA‐0‐week v OA‐12‐weeks*	*OA‐0‐week v OA‐24‐weeks*	*OA‐24‐weeks v OA‐36‐weeks*
Weight (kg)	65.3 ± 12.3	71.0 ± 10.6	70.8 ± 10.1	70.1 ± 10.3	71.0 ± 11.3
	64.9 (20.2)	67.1 (18.2)	66.0 (17.6)	66.1 (18.3)	67.6 (20.8)
		*p = 0.099*	*p = 0.73*	*p = 0.07*	*p = 0.13*
BMI	22.6 ± 2.2	25.0 ± 1.3	24.9 ± 1.2	24.7 ± 1.2	25.0 ± 1.5
	21.8 (3.4)	24.9 (2.4)	24.7 (1.7)	24.6 (1.9)	24.6 (2.5)
		*p = 0.006*	*p = 0.84*	*p = 0.058*	*p = 0.17*
Fat‐mass (kg)	15.3 ± 3.1	22.6 ± 2.8	22.4 ± 2.3	21.7 ± 2.2	
	14.8 (3.5)	22.7 (3.1)	22.3 (3.5)	21.6 (2.6)	
		*p = 0.002*	*p = 0.82*	*p = 0.041*	
Waist circumference (m)	0.78 ± 0.13	0.91 ± 0.08	0.87 ± 0.08	0.86 ± 0.08	0.88 ± 0.09
	0.78 (0.21)	0.90 (0.13)	0.88 (0.11)	0.86 (0.10)	0.88 (0.17)
		*p = 0.01*	*p = 0.0086*	*p = 0.003*	*p = 0.005*
Lean mass (kg)	47.9 ± 12.0	46.3 ± 11.5	46.3 ± 10.9	46.3 ± 11.2	
	48.7 (18.0)	41.5 (21.2)	41.4 (20.3)	41.2 (20.0)	
		*p = 0.61*	*p = 0.96*	*p = 0.85*	

### 3.8 Cognition and physical function

OA had significantly impaired cognition scores on the Montreal cognitive‐assessments, trail making tests A and B, verbal‐fluency test and DSST, together with lower fasting plasma brain derived neurotropic factor (BDNF) level (Table 6) (Figure 2). OA also showed evidence of declining physical function with slower gait speed, lower grip strength, and decreased performance on the rapid 6‐min walk test. GlyNAC supplementation for 24 weeks had a significant impact on both cognitive performance and physical functional tests. GlyNAC significantly improved performance in all measured cognitive functional assessments. In terms of physical function GlyNAC improved the slower gait speed to match YA and also improved handgrip strength in the dominant and nondominant arms, as well as a significant increase in exercise capacity as measured by the rapid 6‐min walk test. However, stopping GlyNAC led to a decline in accrued benefits both for cognitive function and physical function outcomes.

**TABLE 6:** Effect of GlyNAC supplementation and withdrawal on cognition and physical function. OA received GlyNAC supplementation for 24 weeks, and GlyNAC was stopped for 12 weeks from week 24 to week 36. Data are reported as means ± SD and median (IQR). Means are significantly different at p < 0.05

Cognition and physical function	Young adults: 0‐week	Older adults: 0‐week *YA v OA 0‐week*	Older adults: after 12‐weeks on GlyNAC *OA‐0‐week v OA‐12‐weeks*	Older adults: after 24‐weeks on GlyNAC *OA‐0‐week v OA‐24‐weeks*	Older adults: 36‐weeks (12‐weeks after stopping GlyNAC) *OA‐24‐weeks v OA‐36‐weeks*
**Cognition**					
Montreal cognitive assessment test	29.4 ± 0.9	26.0 ± 2.2	28.6 ± 1.4	28.8 ± 0.7	27.5 ± 1.5
	30.0 (1.5)	26.5 (3.5)	29.0 (3.0)	29.0 (1.0)	27.0 (1.5)
		*p = 0.0046*	*p = 0.0007*	*p = 0.0023*	*p = 0.038*
Trails A test (s)	25.2 ± 8.4	45.2 ± 15.1	37.3 ± 10.6	32.1 ± 8.2	33.4 ± 10.4
	25.4 (12.8)	40.0 (22.8)	34.4 (16.8)	30.2 (11.7)	29.5 (12.3)
		*p = 0.004*	*p = 0.012*	*p = 0.003*	*p = 0.58*
Trails B test (s)	36.4 ± 8.7	71.0 ± 26.5	51.5 ± 15.2	48.6 ± 13.4	53.2 ± 23.3
	36.1 (13.1)	72.4 (24.1)	54 (24.8)	47.9 (22.4)	47.0 (31.0)
		*p = 0.007*	*p = 0.023*	*p = 0.016*	*p = 0.38*
Verbal fluency test	52.0 ± 6.7	44.0 ± 10.8	51.4 ± 10.7	52.1 ± 9.7	51.8 ± 13.4
	53.5 (10)	42.5 (14.5)	54.0 (21.0)	51.5 (17.5)	48.5 (18.0)
		*p = 0.059*	*p = 0.0129*	*p = 0.013*	*p = 0.90*
Digital symbol substitution test (% completion)	60.9 ± 9.1	41.3 ± 8.3	42.4 ± 8.2	43.6 ± 8.0	42.1 ± 7.1
	61.4 (13.2)	41.9 (10.5)	44.1 (10.5)	45.5 (8.2)	43.9 (6.4)
		*p = 0.006*	*p = 0.38*	*p = 0.013*	*p = 0.013*
Digital symbol substitution test (% accuracy)	99.4 ± 0.9	96.0 ± 3.1	98.7 ± 2.5	99.6 ± 1.2	97.5 ± 1.4
	100.0 (1.4)	99.6 (3.4)	100.0 (1.9)	100.0 (0.0)	97.7 (1.4)
		*p = 0.018*	*p = 0.0038*	*p = 0.015*	*p = 0.0075*
Plasma BDNF concentration (ng/ml)	36.3 ± 10.6	21.1 ± 4.2	25.3 ± 4.6	31.8 ± 5.6	27.8 ± 5.1
	33.6 (12.9)	21.4 (5.8)	26.5 (5.7)	33.0 (9.5)	26.2 (3.9)
		*p = 0.005*	*p = 0.0002*	*p = 0.0000*	*p = 0.048*
**Physical function**					
Gait speed (m/s)	1.4 ± 0.2	1.0 ± 0.1	1.3 ± 0.2	1.5 ± 0.2	1.2 ± 0.2
	1.4 (0.2)	1.0 (0.2)	1.3 (0.3)	1.5 (0.3)	1.2 (0.2)
		*p = 0.0061*	*p = 0.0007*	*p = 0.0002*	*p = 0.035*
6‐min rapid walk (m)	723.1 ± 66.4	546.4 ± 38.3	578.3 ± 31.6	602.8 ± 43.4	562.1 ± 37.1
	705.0 (80.5)	542.0 (61.5)	578.0 (32.5)	606.0 (65.5)	564.0 (43.0)
		*p = 0.0009*	*p = 0.077*	*p = 0.0074*	*p = 0.0007*
Grip strength, dominant hand (kg)	35.3 ± 5.7	27.0 ± 9.7	29.7 ± 8.7	30.9 ± 9.2	27.3 ± 9.4
	35.4 (9.5)	24.5 (17.9)	26.0 (13.1)	27.2 (16.1)	23.1 (18.3)
		*p = 0.014*	*p = 0.045*	*p = 0.0056*	*p = 0.018*
Grip strength, nondominant hand (kg)	26.0 ± 2.6	23.7 ± 8.4	25.2 ± 7.3	26.6 ± 7.4	24.2 ± 7.6
	26.0 (4.3)	19.9 (14.7)	21.7 (12.5)	22.4 (13.6)	19.9 (14.5)
		*p = 0.013*	*p = 0.08*	*p = 0.004*	*p = 0.0005*

## 4 DISCUSSION

The key findings of this trial are that (1) compared to YAs, OA have GSH deficiency, elevated OxS, impaired MFO, higher inflammation, endothelial dysfunction, insulin resistance, muscle‐protein breakdown, elevated body fat and lower cognitive function, gait speed, muscle strength and exercise capacity, (2) supplementing GlyNAC as GSH precursors for 24‐weeks significantly improved these defects, and (3) accrued benefits receded on stopping GlyNAC for 12‐weeks.

### 4.1 Safety and tolerability of GlyNAC supplementation

GlyNAC supplementation for 24 weeks was well tolerated without any elevation in plasma concentrations of creatinine, alanine transaminase, or aspartate transaminase. On questioning, participants reported no gastrointestinal or any other adverse effects. There were no participant withdrawals from the study.

### 4.2 GlyNAC supplementation improves GSH deficiency and OxS

GSH levels have been reported to be inversely related to multimorbidity in OA.^49^ OAs have an increased prevalence of GSH deficiency.^42^, ^43^, ^44^ GlyNAC supplementation for 24 weeks corrected GSH deficiency, OxS and oxidant damage, but improvement in GSH and OxS reverted to pre‐supplementation levels 12 weeks after stopping GlyNAC, suggesting that continued supplementation of GlyNAC is necessary for maintenance of benefits. However, although F2‐isoprostane levels rose significantly on stopping GlyNAC, they remained much lower than the pre‐supplementation values after 12‐weeks of GlyNAC withdrawal suggesting a long‐term protective effect on oxidative damage could be conferred by GlyNAC. Because this is a pilot study, definitive conclusions cannot be drawn from this observation, but this should be investigated in future studies.

During the process of cellular protection from the harmful effects of OxS, the highly toxic superoxide radical is converted by superoxide dismutase to the less toxic hydrogen peroxide (H_2_O_2_). This process requires complete detoxification of H_2_O_2_ to water, and GSH is necessary for this to occur as depicted in the detoxification reaction H_2_O_2_ + 2GSH → 2H_2_O + GSSG. Thus, GSH adequacy is critically necessary for cellular protection from the ravages of OxS. We reported earlier that GSH deficiency in OA occurs due to diminished synthesis, and this is caused by decreased availability of its precursor amino‐acids glycine and cysteine.^45^ In the same study, we also reported that impaired intracellular RBC‐GSH synthesis, and GSH deficiency can be corrected by supplementing glycine and N‐acetylcysteine (i.e., GlyNAC) for a short duration of 2 weeks. However, the deficiency of glycine and cysteine is in itself intriguing because these are non‐essential amino‐acids which should be synthesized by the body. This study provides clues to understanding the mechanisms underlying their deficiency, and this is discussed in a subsequent section. These unknowns notwithstanding, GlyNAC supplementation in OA provides strong and biologically relevant cellular protection from the harmful and toxic effects of OxS.

### 4.3 GlyNAC supplementation improves mitochondrial dysfunction

Mitochondria generate energy to support life. Aging is associated with mitochondrial dysfunction, which could result in impaired energy availability. In this study, compared to fasting YA, fasting OA had two key defects in fasting MFO – impaired mitochondrial fatty‐acid oxidation and elevated mitochondrial glucose oxidation (MGO). In an earlier study in OA, we found and reported that GlyNAC supplementation for 2 weeks improved mitochondrial fatty‐acid oxidation.^20^ However, the effect of long‐term GlyNAC supplementation on MFO in OA was unknown, especially in relation to the possibility of tachyphylaxis. This study supplemented GlyNAC in OA for a longer duration of 24 weeks and found that the impaired mitochondrial fuel oxidation improved after taking GlyNAC for 12 weeks, without any evidence of tachyphylaxis after 24 weeks of taking GlyNAC. However, the improvements in mitochondrial function were lost on stopping GlyNAC for 12 weeks. These data are consistent with our prior publication that GlyNAC supplementation in aged mice corrected mitochondrial function measured by tracer methodology and molecular techniques.^20^ These results are important and relevant because effective interventions to improve or correct mitochondrial dysfunction in OA (or any human condition) are currently lacking, and GlyNAC supplementation could offer a novel nutritional approach to improve and correct mitochondrial dysfunction in older humans and possibly other human conditions. Additional evidence that GlyNAC supplementation could improve mitochondrial dysfunction in other human conditions comes from our recent report in HIV patients where supplementing GlyNAC at identical doses fully corrected mitochondrial dysfunction.^50^ Although data from this pilot study suggest that GlyNAC can improve mitochondrial function in older humans, this should be confirmed in a randomized clinical trial in older humans.

### 4.4 GlyNAC supplementation improves inflammation and endothelial dysfunction

Chronic, elevated inflammation, and endothelial dysfunction are linked to OxS and to many diseases of aging,^51^, ^52^ but underlying contributory mechanisms are not well understood, and interventions are limited. Consistent with the published literature, this study found that OA had severely elevated inflammation and endothelial dysfunction, but found that these defects improved with GlyNAC supplementation. Data from this trial indicate that GlyNAC supplementation could combat inflammation by its dual actions on lowering elevated levels of pro‐inflammatory cytokines (IL6, TNFα and CRP) and simultaneously increasing the anti‐inflammatory response. It is also interesting to note the measured markers of inflammation and endothelial function do not return to pre‐supplementation values after 12 weeks of withdrawing GlyNAC – this suggests a long‐term persistence of benefits due to GlyNAC. The mechanisms to account for this are not clear and should be investigated in future studies.

Because inflammation, endothelial dysfunction, insulin resistance, mitochondrial dysfunction, and increased waist circumference are linked to an elevated risk of metabolic syndrome and cardiovascular disease in aging humans, improvements in these defects with GlyNAC supplementation suggest the possibility of promoting cardiovascular and cardiometabolic health. Evidence to support this comes from our recent publication where GlyNAC supplementation in aged mice was found to correct defective cardiac mitochondrial energetics, lower cardiac inflammation, and improve diastolic dysfunction.^53^ Further studies are needed to understand the impact and implications of GlyNAC supplementation on lowering inflammation, endothelial dysfunction, and cardiometabolic risks in aging humans.

### 4.5 GlyNAC supplementation lowers insulin resistance

GlyNAC supplementation significantly lowered insulin resistance in OA. Elevated OxS has adverse effects on insulin signaling^54^ and Glut4 transcription,^55^ and promotes inflammation^56^ and mitochondrial dysfunction.^57^, ^58^, ^59^ The improvement in OxS, inflammation and mitochondrial dysfunction in this study could have contributed to the observed significant decrease in insulin resistance.

### 4.6 GlyNAC supplementation improves cognition

Aging is the greatest risk factor for cognitive decline and dementia affecting millions of OA and results in impaired learning, memory, executive function, attention, language, perceptual motor function, and social cognition,^59, 60^ but effective interventions are either limited or lacking.

Glucose is the primary fuel for the brain. In this study, we found two paradoxical observations regarding whole‐body MGO and cognition as measured by testing performance: (1) prior to starting GlyNAC supplementation, OA had impaired cognition (compared to YA) despite increased whole‐body MGO and (2) after GlyNAC supplementation their whole‐body MGO decreased, but cognition improved. The most parsimonious explanation for these opposing and paradoxical findings is as follows: In the fasting state, glucose provides energy for the brain, and fatty‐acids provide energy for non‐brain tissues. GSH adequacy is essential for optimal mitochondrial fatty‐acid oxidation.^20^ OA in this study had GSH deficiency, impaired whole‐body mitochondrial fatty‐acid oxidation and higher whole‐body MGO prior to GlyNAC supplementation suggesting that non‐brain tissues were using glucose instead of fatty‐acids for energy needs. However, tracer studies indicated that the glucose production rates in OA were not increased compared to YA and did not change with GlyNAC supplementation, suggesting that the available glucose pool remained constant. In this situation where the glucose pool available for energy needs has not expanded, any increase in glucose consumption by non‐brain tissues will lead to diminished glucose availability for the brain. We term this the "glucose‐steal phenomenon" where non‐brain organs by virtue of switching to glucose oxidation are competing for glucose with the brain. This "glucose‐steal" could explain the paradox of increased whole‐body MGO, but lower cognitive performance. With GlyNAC supplementation, the non‐brain tissues revert to using fatty‐acids as a source of fuel and relinquish utilization of glucose – this results in a fall in the magnitude of whole‐body MGO, and more glucose is now available for the brain (a reversal of the "glucose‐steal phenomenon"), and cognitive improves. Elevated insulin resistance could also impact cognition by limiting glucose entry into the brain, and improvement in insulin resistance has been linked to improved cognition.^61^ OA in this study had severely elevated insulin resistance which improved significantly with GlyNAC supplementation and could have contributed to cognitive improvement. The dual impacts of glucose‐steal and elevated insulin resistance in this study could help explain why FDG‐PET scans show low glucose uptake in cognitively impaired OA.^62^ Because cognitive impairment is also associated with elevated brain inflammation and endothelial dysfunction,^63^ improvements in these defects with GlyNAC supplementation could also have contributed to cognitive improvement in this study. GlyNAC could also be having a direct effect in the brain as seen by a significant increase in the diminished levels of the brain biomarker BDNF. BDNF is involved in the preservation of memory, synaptic plasticity, and maintenance of neuronal networks,^64^, ^65^ and lower plasma levels of BDNF are associated with lower cognitive test scores and mild cognitive impairment.^66^ In this study, OA with lower cognitive scores had lower levels of BDNF, which improved significantly in parallel with the cognitive improvement with GlyNAC supplementation, and fell on stopping GlyNAC. The results of the study do not support that cognitive improvements may occurred due to practice effects as a result of repeated testing. The basis of the practice effect is that test scores improve with repeated test taking. The cognitive test scores of OA did improve between the first time it was administered (0 weeks), midpoint (12 weeks) and end of supplementation (24 weeks). However, when it was taken a 4th time, 12 weeks after stopping supplementation (at 36 weeks), several scores declined, which argues against a learning/practice effect. Changes in BDNF concentrations which track closely with cognitive changes in this study as a result of GlyNAC supplementation and withdrawal also argue against a practice effect. Collectively, these observations offer early exciting leads which suggest that GlyNAC supplementation could potentially improve cognitive function in OA. Although this is a pilot trial, these findings could be relevant because cognitive impairment in OA is not well understood, and there are no effective interventions, and results support the need for future trials focused on GlyNAC to understand its impact on cognition as well as underlying mechanistic defects linked to cognition in terms of OxS, GSH deficiency, mitochondrial impairment, inflammation, insulin resistance, and endothelial function.

### 4.7 GlyNAC supplementation improves muscle protein breakdown – implications for sarcopenia

Maintaining optimal muscle mass depends on a balance between muscle protein synthesis and breakdown. In the fasting state, muscle protein breakdown exceeds synthesis,^67^ which results in net protein loss. Sarcopenia in OA involves both declining muscle mass and declining muscle strength. Combating sarcopenia is restricted to boosting muscle protein synthesis, due to lack of interventions to lower muscle protein breakdown. Therefore, our finding that GlyNAC supplementation decreases muscle breakdown could have important implications for sarcopenia. However, muscle mass did not improve with GlyNAC, suggesting that combining dual strategies to boost muscle synthesis and also decrease breakdown may be necessary for combating muscle loss in sarcopenia. Although there was no increase in muscle mass, GlyNAC supplementation was associated with a significant improvement in muscle strength and physical function, and this is discussed next.

### 4.8 GlyNAC supplementation improves gait speed, strength, and exercise capacity

Increasing age is associated with physical decline and diminished exercise capacity, both of which are components of sarcopenic obesity in OA. Although a key meta‐analysis in OA has shown that lower gait speed is linked to decreased survival,^68^ restoring gait speed in OA to levels seen in YA has been difficult to achieve. This study found that GlyNAC supplementation for 24 weeks increased gait speed in OA to match YA while simultaneously improving muscle strength and exercise capacity, suggesting that GlyNAC supplementation improves physical function in OA. While the underlying mechanisms contributing to the improvement in physical function are not clear, it is known that mitochondrial function is linked to strength and walking speed.^69^ Therefore, mitochondrial dysfunction could have contributed to the decline in muscle strength and physical function observed in OA prior to GlyNAC supplementation, and the improvement in mitochondrial dysfunction with GlyNAC could have played a role in the observed improvement in muscle strength and physical function.

### 4.9 GlyNAC supplementation improves body composition

Although impaired MFO is a risk factor for weight gain,^70^ it is unclear whether improving MFO can induce weight loss. This study found that improving MFO was associated with a significant reduction in total body fat and waist circumference, which suggests that the fat loss may have preferentially occurred in the abdomen.

### 4.10 Genomic damage and cancer risk

Aging is associated with genomic damage, which is an aging hallmark, and is linked to cancer. In this study we measured and found that compared to YA, OA had significantly higher levels of plasma 8‐OHdG (8‐hydroxy‐deoxyguanosine), a validated biomarker of DNA (deoxyribonucleic acid) damage. Both increased ROS (which induce OxS) and 8‐OHdG have been linked to increased risk of developing colon, prostate, and breast cancer.^71^, ^72^, ^73^, ^74^ This trial finds that supplementing GlyNAC in OA significantly lowers biomarkers of OxS, damage due to OxS and 8‐OHdG, and that these outcomes increase on stopping GlyNAC. However, whether GlyNAC can lower cancer risk in humans is unknown and needs to be evaluated in future studies. Metabolic profiling has identified a possible role for glycine in cancer cell proliferation,^75^ but glycine supplementation was found to prevent the development of hepatic tumors^76^ in rats, inhibit the growth of melanoma tumors^77^ in mice, and to also inhibit tumor growth in a mammary adenocarcinoma cell line.^78^ A more recent study from the National Institutes of Health (NIH) Intervention Testing Program reported that mice supplemented with glycine lived 4%–6% longer and had a lower presence of pulmonary adenocarcinoma.^79^

### 4.11 Mechanism for deficiency of glycine and cysteine in aging

Because cysteine and glycine are gluconeogenic amino‐acids, could they be used for increasing gluconeogenesis to support the abnormally elevated fasting MGO? Tracer data refute this as glucose production rates were not increased in OA, therefore glycine and cysteine were not being used for glucose production. The most parsimonious explanation is that due to a severe defect in MFO, amino‐acids (including glycine and cysteine) were being diverted toward energy generation. This explanation could also account for the elevated muscle protein breakdown seen in OA and its recovery with GlyNAC supplementation in OA.

### 4.12 Potential implications for defects in aging hallmarks

The field of geroscience has identified at least nine hallmark defects of aging which are believed to contribute to the aging process.^80^ These hallmark defects of aging include mitochondrial dysfunction, dysregulated nutrient sensing (which includes insulin resistance), altered intercellular communication (which includes inflammation), genomic instability, loss of proteostasis, epigenetic alterations, cellular senescence, telomere attrition, and stem cell exhaustion. Interventions to correct these aging hallmark defects in older humans are either limited or lacking. Therefore, it is exciting that this study finds that GlyNAC supplementation appears to improve the components of four aging hallmarks (i.e., improvements in mitochondrial dysfunction, inflammation, insulin resistance and genomic damage) and warrants further study in a randomized clinical trial.

### 4.13 Why does GlyNAC work? *The "power of 3"*

This is an important question because of the breadth and depth of improvements in multiple parameters, especially when a single nutritional intervention appears to correct *all* of these defects. We speculate that GlyNAC represents three forces which could be operating simultaneously to result in such widespread improvements: (1) Correction of GSH deficiency, which results in correction of OxS and mitochondrial dysfunction. GSH is the most abundant endogenous, intracellular antioxidant. Any intervention to correct GSH needs to necessarily correct intracellular GSH concentrations, and GlyNAC does this efficiently and effectively. However, correction of GSH alone is insufficient to explain the magnitude and extent of improvements, suggesting that there may be other factors in play; (2) GlyNAC contains glycine, an important methyl‐group donor. Methyl groups are abundant in DNA and are important components of multiple cellular reactions. Glycine is also important for normal brain function. Hence providing glycine could improve multiple defects as seen in this trial; (3) GlyNAC contains N‐acetylcysteine, which functions as a cysteine donor. Cysteine is critically important in energy metabolism by contributing the sulfhydryl (SH) group needed for energy generation. For example, coenzyme A (CoA‐SH) is an important component of reactions governing energy generation and depends on the availability of a SH group for its normal functioning. Cysteine and its donated SH groups also play key roles in multiple additional cellular reactions and function.

Thus, it can be seen that the combination of glycine, cysteine, and GSH could be acting in combination to result in powerful improvement, recovery, and correction of multiple abnormalities and defects as seen in this trial in older humans, and results in increased strength and improved cognition. This could explain why GlyNAC supplementation works to promote healthy aging.

### 4.14 Study limitations

Although the observations in this study support the impact of supplementing GlyNAC on improving several age‐associated defects, these results should be viewed with the caveat that this is a pilot trial, the study sample size was small, and that the study lacked a blinded placebo group of OA. Nevertheless, these results suggest that GlyNAC supplementation improves cellular protection, mitochondrial energy metabolism, strength, physical function, and cognitive health in OA. The rapidly increasing world population of older humans will result in an urgent need for novel strategies to promote healthy aging to prevent diseases stemming from age‐associated metabolic and mitochondrial abnormalities, and therefore the results of this open‐label trial supports the need for additional trials of GlyNAC supplementation in aging.

## 5 CONCLUSIONS

The results of this trial suggest that GlyNAC supplementation in OA is well tolerated and could play a novel role in improving healthy aging in OA by correcting GSH deficiency, OxS, mitochondrial dysfunction, inflammation, insulin resistance, endothelial dysfunction, body fat, muscle strength, gait speed, and cognitive function. GlyNAC supplementation also appears to improve hallmark defects in aging, which could be an exciting discovery and needs to be confirmed in future studies. Dietary supplementation of GlyNAC could improve the cellular, mitochondrial, and metabolic health of OA, improve strength and cognition, and thereby promote healthy aging. No side‐effects of GlyNAC supplementation were detected during 36‐week duration of the study. The results of this study support the need for future trials to assess the effects of GlyNAC supplementation in a larger population of OAs receiving GlyNAC supplementation for a longer duration.

## CONFLICTS OF INTEREST

None of the authors have any conflicts of interest to disclose.

## AUTHOR CONTRIBUTIONS

Premranjan Kumar performed blood analyses and contributed to manuscript preparation. Chun Liu was the study coordinator and processed tracer samples. Charles Minard performed statistical analyses. Shaji Chacko designed the glucose‐tracer protocol and performed glucose‐tracer calculations. Jean W. Hsu prepared tracer solutions and performed methylhistidine‐tracer calculations. Farook Jahoor designed the methylhistidine‐tracer protocol, supervised Jean W. Hsu and Chun Liu, and contributed to the manuscript. Rajagopal V. Sekhar conceived the hypotheses, designed the trial, obtained funding, developed the GlyNAC intervention, recruited and consented subjects, supervised Premranjan Kumar and Chun Liu, interpreted data, and wrote the manuscript.

## FUNDING INFORMATION

This work was supported by a philanthropic gift from the McNair Medical Institute at the Robert and Janice McNair Foundation in Houston, Texas, USA.

## Supporting information

Supporting Information

## Data Availability

Data will be made available on reasonable request.
