# Supplementary figures and images for: Glycine and N‐acetylcysteine (GlyNAC) supplementation in older adults improves glutathione deficiency, oxidative stress, mitochondrial dysfunction, inflammation, insulin resistance, endothelial dysfunction, genotoxicity, muscle strength, and cognition: Results of a pilot clinical trial
Source: Clin Transl Med. 2021 Mar 27;11(3):e372. doi: 10.1002/ctm2.372 (PMC8002905; doi:10.1002/ctm2.372)

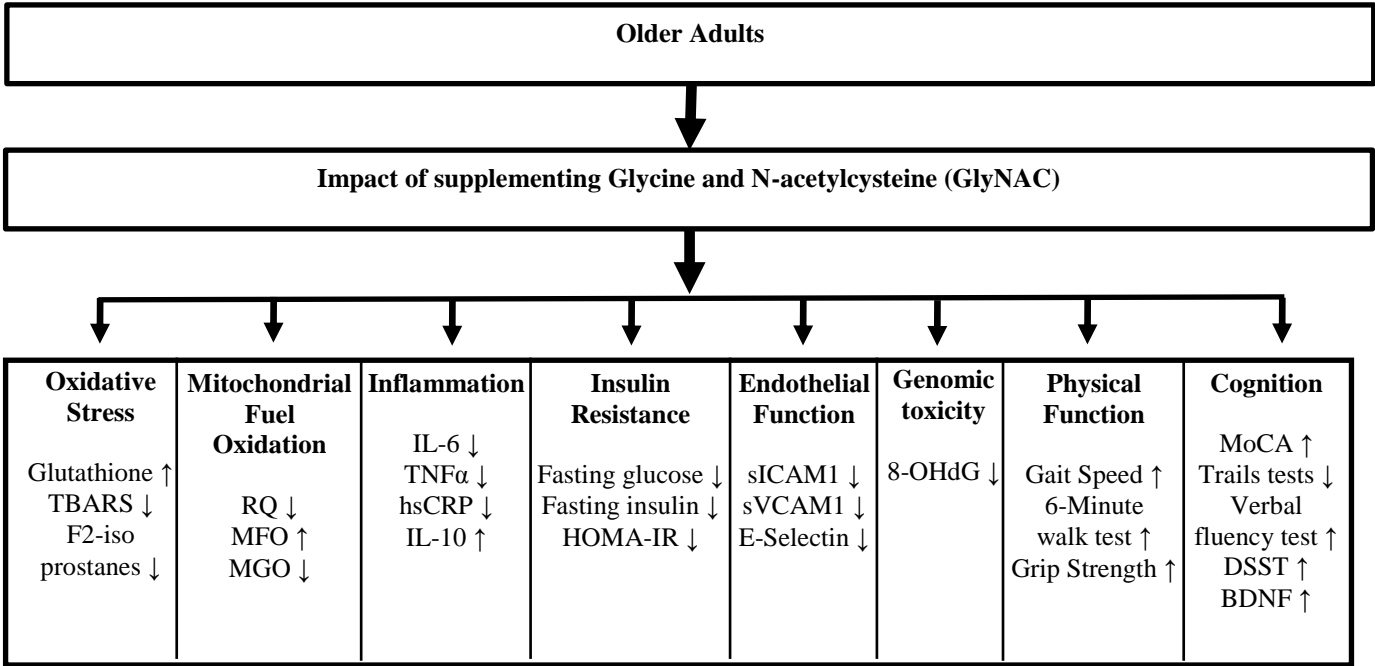

Supplement: Supplementary file 1 — Supporting Information [file CTM2-11-e372-s001.pdf]
